# Use of supporting evidence by health and industry organisations in the consultation on e-cigarette regulations in New Zealand

**DOI:** 10.1371/journal.pone.0275053

**Published:** 2022-09-29

**Authors:** Lucy Hardie, Judith McCool, Becky Freeman

**Affiliations:** 1 School of Population Health, Faculty of Medical & Health Sciences, University of Auckland, Auckland, New Zealand; 2 School of Public Health, The University of Sydney, Sydney, New South Wales, Australia; Shenzhen University, CHINA

## Abstract

**Objectives:**

Scientific evidence to support the development of appropriate policy for electronic cigarette use is limited by rapidly changing technology and a lack of long-term data. Perceptions of risk and benefits determine diverse framings of the e-cigarette debate and complicate policy decisions. E-cigarette use by smokers who are attempting to quit may result in improved health outcomes, while their use among young people and non-smokers may lead to adverse health consequences. The purpose of this study was to identify the types of evidence used during public consultations on proposed revisions to New Zealand’s e-cigarette legislation in 2020.

**Methods:**

Using submissions to parliament made by the tobacco/e-cigarette industry and the health sector, we assessed the cited evidence for quality and independence measured by publication type and tobacco industry connections. We identified themes from a sub-sample of *frequently cited evidence* to understand how stakeholders and organisations used evidence.

**Results:**

The sample consisted of 57 submissions from the e-cigarette and tobacco industry (*n* = 21) and health organisations (*n* = 36). A total of 442 pieces of evidence were cited at least once. Health organisations were more likely to cite peer-reviewed evidence (*OR =* 2.99). The industry was more likely to cite evidence outside of peer review and sources with tobacco industry connections (*OR =* 4.08). In the sample of frequently cited evidence, *youth prevalence* and *flavours* were the most common themes. In some cases the same evidence was used by both groups to support opposing policy positions.

**Conclusions:**

The industry continues to rely more heavily on evidence published outside of the peer-review process, which is, therefore, subjected to less scientific scrutiny. By using a smoking-cessation or harm-reduction narrative, the industry could be seen as a legitimate stakeholder in policy development.

## Introduction

Health policies informed by high quality, rigorous, and systematic evidence are more likely to be effective [1]. Evidence-informed policymaking attempts to move from ideological or value-based decision making towards the inclusion of substantiated and objective knowledge. Scientific literature on electronic cigarettes (e-cigarettes) is emerging, but is limited by a lack of long-term data on health impacts, and rapid changes in key design features meaning research becomes quickly outdated [2]. Whilst a range of viewpoints exist on the regulation of e-cigarettes [2], the process has been complicated by some polarisation of e-cigarette framing in research focus and policy approaches [3, 4]. One such approach emphasises the harm reduction potential of e-cigarettes as a replacement for smoking, whilst another focuses on concerns that e-cigarettes enable the tobacco industry to exploit and addict new consumers [5, 6]. Both perspectives claim their views are built on robust evidence [6].

The New Zealand Government stated its intention to regulate e-cigarettes in 2017 [7]. However, nicotine e-cigarettes were treated as prohibited in New Zealand [NZ] until March 2018. It was at this time that a court ruling (Philip Morris International vs Ministry of Health) reasoned that New Zealand’s existing tobacco legislation did not include aerosol products (e-cigarettes). [8]. Although the case involved heated tobacco products, the ruling was interpreted as being applicable to all electronic cigarettes. Following this ruling, the NZ government initiated a consultation process to update the existing legislation to regulate e-cigarette products. The Smokefree Environments and Regulated Products (Vaping) Amendment Bill, 2020 (hereafter, the Bill) was presented in March 2020, allowing e-cigarettes to be openly sold and marketed in NZ for nearly two years with few restrictions.

During this period, industry marketing efforts intensified [9], and e-cigarette use increased significantly, especially among young New Zealanders (15–24 year olds). The rate of regular use (at least once a month) increased from 4.9% in 2018 to 18.5% in 2020 [10].

As part of the Bill’s consultation process, the government called for public submissions for consideration by the Health Select Committee. Despite a nationwide COVID-19 lockdown during this period, there was a great deal of interest in the Bill, with over 1,200 written and 84 oral submissions. Our study examines the research evidence cited by selected stakeholders to support policy positions and claims on the proposed regulation of e-cigarettes in New Zealand. Health organisations and industry organisations, including e-cigarette and tobacco companies (hereinafter "the industry"), were considered to have the greatest interest in this policy debate. Health organisations serve populations of interest and have relevant public health and scientific expertise. The industry has considerable financial investments, resources, and e-cigarette product knowledge. Therefore, both groups are well positioned to provide comments on the proposed legislation. Aligned with previous research [11] we assessed the quality of evidence, by publication type, the most frequently cited evidence, and whether cited evidence was connected to the tobacco industry.

## Methods

### Sample identification

**Fig 1:**
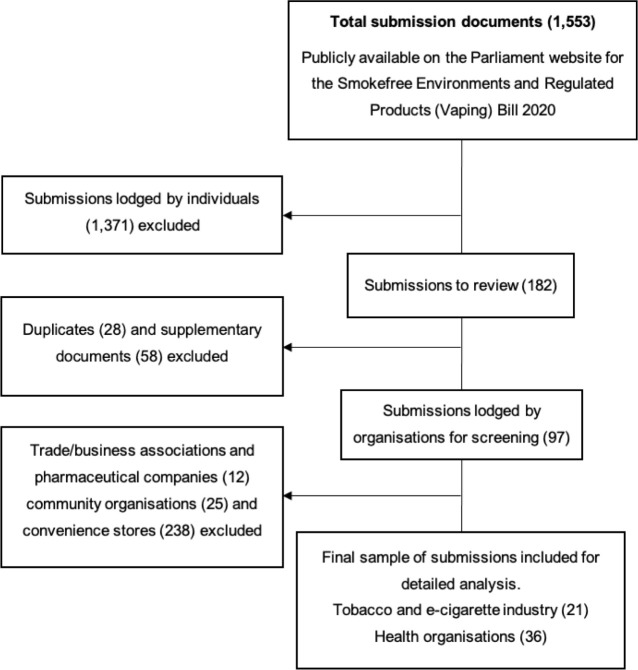
Sample identification procedure.

Written submissions on the proposed Bill, made by organisations, were obtained from the New Zealand Parliament website [12]. We identified 21 submissions by the e-cigarette and tobacco industry and 36 submissions made by health organisations for analysis (Fig 1). The ‘industry’ was defined as submissions made by tobacco and dedicated e-cigarette retailers, manufacturers and distributors [13] including organisations funded directly or indirectly by the tobacco industry [14]. ‘Health organisations’ were defined as submissions made by health-focused non-government organisations, medical associations, research groups affiliated with recognised tertiary education providers and organisations that deliver health services. Submissions that did not meet these criteria (Fig 1), and supplementary documents, which are often large and not directly related to the policy, were excluded. The complete list of included organisations and their definitions is included in S1 File.

### Coding and analysis

#### Type of publication

All citations included in the sample of submissions were imported into Microsoft Excel 16.47.1. We defined a piece of evidence as a unique work or publication. To assess the quality of the cited evidence, each piece was coded by publication type as outlined in Table 1. The statements made by organisations which the cited evidence supported were also recorded. Chi-square tests (*p =* .05) were used to evaluate differences between organisation type and publication type using SPSS 27.

**Table 1:** Coding framework for quality of evidence.

Coding framework for classification of quality of evidence		
Criteria	Coding framework	Categories
Quality	*What type of publication was used as cited evidence*?	**Peer-reviewed/Academic** ◦ Peer-reviewed journal articles, systematic reviews & peer-reviewed books
		**Reports** ◦ Based on academic research ◦ NZ government reports ◦ UK government reports ◦ Privately commissioned publication
		**News Articles** ◦ News/broadcasting organisations, e.g. TVNZ, CBS
		**Websites** ◦ Industry/retail websites, e.g. Vype, vaping360 ◦ Organisation websites, e.g. Asthma Foundation ◦ Information websites, e.g. Ministry of Health
		**Other** ◦ Video recordings, presentations, letters, blogs
Independence	*Is the evidence independent from the tobacco industry*?	**Independent of the tobacco industry** ◦ No tobacco industry connections in either authorship or funding
		**Tobacco industry connected** ◦ Source funded by the tobacco industry, e.g. research grants, commissioned reports ◦ An author employed by or has received benefits from the tobacco industry

Table adapted from Hatchard et al. [11]

#### Tobacco industry connections

To assess potential industry influence, we identified tobacco industry connections for all the extracted publications according to the criteria in Table 1. We appraised the declared funding sources, acknowledgements, and conflicts of interest to determine associations with the tobacco industry (*n =* 33).

#### Frequently cited evidence

We determined the most frequently cited sources based on the number of organisations, within each group, that cited each piece at least once. We ranked each source by the number of organisations that cited each piece and included the ten most frequently cited pieces for each group. We then conducted a thematic analysis of the ten most frequently cited sources for each of the two groups (*n* = 19).

#### Main themes from frequently cited evidence

Based on the statements each citation supported, we generated codes iteratively from the subset of frequently cited evidence. Coding was developed by identifying common themes in the text, which were refined and reviewed by the authors. Table 3 describes how statements were categorised according to the finalised codes. For example, the statement *“no reputable epidemiologist or scientific evidence in New Zealand has found evidence of a youth vaping epidemic”* was coded under ‘e-cigarette prevalence among young is negligible’. The first author referred to the original submission when additional context was needed to accurately code each statement. A subset of 30 randomly selected statements was coded by each author in order to ensure consistency. Having reached 93% agreement, the first author analysed the remaining content. S1 File contains the complete list of statements.

## Results

### Sample

A total of 57 submissions were included in the sample consisting of 21 submissions made by the industry and 36 made by health organisations (Fig 1).

### Quality of sources cited and source independence from the tobacco industry

Four hundred and forty-two pieces of evidence were cited at least once in the total sample of submissions (*n =* 57). As shown in Table 2, the proportions of the types of evidence cited differed significantly between the industry and health organisation submissions (*x^2^* (4, *n =* 464) = 27.651, p<0.001). Industry lodged submissions cited a significantly lower proportion of peer-reviewed evidence (37%) compared to health organisations (64%). Industry submissions were significantly more likely to cite reports, news articles or other sources. On a per submission basis, health organisations were three times as likely to cite peer-reviewed evidence with an average of 9.6 peer-reviewed sources per submission, compared to 2.1 per industry submission. The industry was more likely to draw upon evidence connected to the tobacco industry (15.5%) compared with 4.3% of health organisations. Eleven of the 18 tobacco industry connected sources presented by industry were peer-reviewed. Of the tobacco industry connected evidence cited by health organisations, the majority (67%, *n = 10*) were utilised by one health organisation. The majority of supporting evidence in the *industry* group was provided by tobacco companies (91%); independent e-cigarette companies cited just 11 of the 116 publications for this group.

**Table 2:** Publications cited in submissions by Industry and health organisations.

Evidence type	Industry submissions (*n* = 21)		Health organisation submissions (*n* = 36)		Odds ratio	95% CI
	Publications (*n* = 116)	%	Publications (*n* = 348)	%		
***Quality***						
Peer-reviewed publications	43	37%	222	64%	0.33*	[0.22 – 0.52]
Reports	33	28%	64	18%	1.76*	[1.09–2.87]
Websites	14	12%	27	8%	1.63	[0.82–3.23]
News articles	8	7%	8	2%	3.15*	[1.15–8.59]
Other	18	16%	27	8%	2.18*	[1.15–4.13]
***Independence***						
Tobacco industry connected	18	15.5%	15	4.3%	4.08*	[1.98–8.39]

*p <0.05

### Frequently cited evidence

The most frequently cited evidence utilised by health organisations were peer-reviewed publications (*n =* 7), followed by reports (*n =* 2) and one academic blog. The most frequently cited evidence utilised by industry were reports (*n =* 6), followed by peer-reviewed publications (*n =* 3), and the Bill itself. One peer-reviewed journal article (Walker et al. 2020) was cited by 14 health organisations and two industry organisations, making it the most cited piece of evidence in the sample. A summary of the most frequently cited sources is provided in S1 File.

### Main themes from frequently cited evidence

From the subset of most *frequently cited evidence*, central themes were identified. The most common themes were youth prevalence (*48 citations using 3 pieces of evidence)* and flavours (*40 citations using 5 pieces of evidence)*. These themes mapped closely to key policy areas addressed within the proposed Bill. We appraised how both the industry and health organisations utilised these pieces of evidence in their submissions.

### Use of evidence related to youth e-cigarette use and smoking

The following sources of youth-prevalence related evidence were most frequently cited: Walker et al. 2020 [15], ASH NZ, 2019 [16] and Ball et al. 2020 [17]. This set of evidence was used to support two distinct positions, shown in Table 3. The first position aligned with a *smoking cessation focus*, and supported statements made by organisations that e-cigarette use was supporting a reduction in youth smoking, and/or that e-cigarette use among youth is negligible. The second position aligned with a *youth and non-smoker focus* and supported claims that youth uptake of e-cigarettes is high, and/or that youth smoking rates are increasing. Our analysis indicates that of the organisations that cited these three documents, the industry aligned with the *smoking cessation focus*, as did four of the health organisations, while the remaining 14 health organisations that used these sources to support their position aligned with the *youth and non-smoker focus* (S1 File).

**Table 3:** Number and position of organisations that utilised frequently cited evidence relating to ‘youth prevalence’ and ‘flavours’.

**Youth e-cigarette and smoking prevalence** *Walker et al*. *2020*, *ASH NZ*, *2019 and/or Ball et al*. *2020*			
Theme	Industry (*n =* 3)	Health (*n =* 18)	Examples
**Smoking cessation focus** • e-cigarette use is supporting a reduction in youth smoking • e-cigarette prevalence among youth is negligible	3	4	*“Indeed*, *in New Zealand*, *evidence suggests that e-cigarettes might be displacing smoking”*—Tobacco Transnational Company *“The findings concluded that there was no vaping epidemic*, *and vaping may be displacing smoking in young people”*–Health-oriented NGO
**Youth and non-smoker focus** • e-cigarette prevalence among youth is high, and/or • youth smoking rates are increasing	0	14	*“We note that there is emerging evidence that the recent increase in vaping among school students (nearly 40% had tried vaping in 2019*, *and 12% were vaping regularly has been accompanied by an increase in regular youth smoking*.*”*–Public Health Service *“Significantly*, *sixty five percent of students who had ever vaped and forty eight percent of those who regularly vaped had never smoked cigarettes”–*Stop Smoking Service
**E-cigarette flavours** Russell et al. (2018), Farsalinos et al. (2013), Gendall et al. (2020), Zare et al. (2018) and/or Meernik et al. (2019)			
Theme	Industry (*n =* 2)	Health (*n =* 13)	Examples
**Smoking cessation focus** • flavours are important for smoking cessation and/or • restrictions could have unintended consequences for smokers, e.g., relapse	2	2	*“Research suggests over-regulation may encourage NGP users*, *particularly vapers*, *to concoct DIY flavours*.*”* –Tobacco Transnational Company *“the study concluded that restricting the availability of e-cigarette flavours could reduce adult smokers’ interest in switching to e-cigarettes and raises the possibility that e-cigarette users could return to combustible tobacco products*.*”* –Tobacco Transnational Company
**Youth and non-smoker focus** • e-cigarette flavours increase appeal beyond smokers, (non-smokers/young people) and/or • flavours decrease harm perceptions	0	11	*“Flavours may be the most important reason for adolescents trying e-cigarettes*.*”* –District Health Board *“Systematic reviews suggest that flavours increase product appeal*, *decrease harm perception*, *and may be the most important factor in young people trying e-cigarettes*.*”* –Health-oriented NGO

### Use of evidence to support claims related to e-cigarette liquid flavours

Five of the most frequently cited sources related to *e-liquid flavours* and included: Russell et al. (2018), Farsalinos et al. (2013), Gendall et al. (2020), Zare et al. (2018) and Meernik et al. (2019). Two distinct positions were again identified. The first position aligned with the *smoking cessation focus*, that flavours are important for smoking cessation, and/or any restrictions could have unintended consequences for smokers, e.g., relapse. The second position aligned with *the youth and non-smoker focus* and supported statements that flavours increase appeal beyond smokers to non-smokers and/or young people and/or that flavours decrease perceptions of harm. Of the organisations to reference any of the frequently cited pieces, the industry *(n = 2)* aligned with the *smoking cessation focus*, as did two health organisations and the remaining 11 aligned with the *youth and non-smoker focus* (S1 File).

## Discussion

Our study was designed to assess the types of evidence presented by key stakeholders in the consultation on e-cigarette regulations. Findings demonstrate that health organisations were more likely to cite peer-reviewed, *independent* publications compared to industry organisations. Peer-review evidence is generally considered more reliable and robust than sources that have not been through the same scrutiny [1, 18]. Our findings are consistent with previous research demonstrating that the industry regularly presents evidence with less scientifically rigorous methods to support its policy arguments [19]. The industry group also drew on more tobacco industry-funded sources, consistent with previous research findings [19, 20]. Whilst smaller e-cigarette companies in this study provided a small portion of supporting evidence compared to tobacco companies, research demonstrates that e-cigarette retailers in NZ regularly present scientific claims in promotional material [21] and therefore should have this data available to cite.

Our study reveals that the same pieces of evidence were used to support divergent policy positions. Using the same evidence, but arriving at a different conclusion, is not uncommon within the e-cigarette policy debate [6, 22]. For example, evidence reviews conducted by both Public Health England and the National Academies of Science, Engineering and Medicine presented broadly consistent findings, however, the organisations came to fundamentally different policy positions [6]. These divergent positions appear to result from the value-based prioritisation of key populations, either those who use e-cigarettes for smoking cessation, or young people vulnerable to e-cigarette and smoking uptake [3, 22, 23]. Three pieces of evidence in our study were cited to both support and refute claims that the wide availability of e-cigarettes in NZ has contributed to considerable youth uptake or increased youth smoking rates. Our study shows that of the 18 health organisations that cited at least one of the three pieces of *frequently cited* relating to *youth*, most (*n =* 14) presented concerns about e-cigarette use among youth in NZ.

The industry’s statements in the sub-set of *frequently cited evidence* are positioned within a smoking cessation focus. For example, one tobacco company stated, “the removal [of e-cigarette flavour options] will likely cause some smokers to relapse” [24]. This approach may position the tobacco industry as part of the solution to smoking related health impacts, serving as a mechanism to rebuild a tarnished reputation. However, internal industry documents reveal that the marketing of e-cigarettes is part of strategic plans for developing new markets [25, 26] while retaining a core business focus in combustible cigarettes [27]. This position supports the tobacco industry’s interests in growth within the global market, renewing market viability in countries where smoking rates are declining. The importance of these new markets is becoming increasingly clear as the tobacco industry-owned global e-cigarette share has increased by 25% to 43.6% in the previous five years [27]. This market share includes the tobacco industry acquisition of a large stake in the brand JUUL which dominates 40% of the US market and is synonymous with youth vaping [28].

While it is expected that stakeholders use evidence that will support their policy preferences, the use of tobacco-industry connected sources is highly problematic. Our study reveals that despite the availability of the latest New Zealand specific evidence on flavours [29] and two recent systematic reviews in this field [30, 31], the industry opted instead to utilise one tobacco industry funded publication [32] and one publication from 2013 [33] to support their policy positions in this area. It has been widely acknowledged that the tobacco industry has sought to shape the evidence base by funding research and our findings confirm that industry-led submissions cited evidence that concur with industry aims [34, 35]. Previous research shows that a conflict of interest is associated with results more favourable to industry objectives of increased market growth. For example, fewer health impacts of e-cigarettes are reported when compared with independent studies [36]. For this reason, leading health journals refuse to publish tobacco industry-funded research [37, 38]. The World Health Organization (WHO) acknowledges that the main obstacle in the effort to curb tobacco use globally is the tobacco industry, and reiterates that, “There is a fundamental and irreconcilable conflict between the tobacco industry’s interests and public health policy interests” [13, 39]. In alignment with the WHO FCTC, of which New Zealand is a signatory, article 5.3 requires parties to protect public health policy from interference by the tobacco industry [40]. Although tobacco companies are required to disclose their affiliations in New Zealand parliamentary submissions [41], this process may enable the tobacco industry to engage in the policy process undermining the WHO FCTC [20, 42].

## Limitations

Our study has some limitations. Conflicts of interest are self-defined and operate on a trust-based model where authors declare their interests and funding sources truthfully. However, the enforcement of these conflicts is limited [22], and therefore the influence of the tobacco industry in the evidence base may be higher. E-cigarette industry conflicts were not assessed. Supplementary documents included with the submissions were removed and may have contained further citations impacting the results. While we reported on health organisations’ use of tobacco industry evidence, three of the citations by health organisations were to highlight tobacco industry tactics rather than support claims on e-cigarettes for smoking cessation. In the sample of *most frequently cited* evidence some organisations did not cite these sources but did argue a position on *flavours* or *youth uptake*. It should also be noted that this study uses publication type as an indicator for quality of evidence, but findings do not include an in-depth quality assessment for each piece. We acknowledge that standards vary in peer-review [43] and other publication types. However, our study presents a method that may be useful for policymakers identifying potential aspects for consideration when assessing large amounts of evidence.

## Conclusion

E-cigarette regulation globally is evolving as jurisdictions seek to adopt regulations for new products. Policy makers look to evidence to inform decisions, yet our study shows that given the emerging and changing nature of e-cigarette evidence, the very same research can be positioned by opposing interests to support both stronger and weaker regulations. Further, the industry utilises more evidence published outside of the peer-review processes, subject to less scrutiny from scientific experts, and evidence linked to the tobacco industry compared to health organisations. The industry prioritises a smoking cessation framing to gain acceptance in the policy process and positions itself as part of the solution to smoking-related disease. Signatories of the WHO FCTC are expected to protect public policy from tobacco industry interference and must treat industry evidence with caution. A process of organising evidence by publication type and independence may benefit policymakers to prioritise higher quality evidence and identify that which may require further scrutiny, particularly publications with tobacco industry connections.

## Supporting information

S1 File Study sample, frequently cited evidence & main themes. (PDF)
