# Supplementary material for: Use of supporting evidence by health and industry organisations in the consultation on e-cigarette regulations in New Zealand
Source: PLoS One. 2022 Sep 29;17(9):e0275053. doi: 10.1371/journal.pone.0275053 (PMC9522304; doi:10.1371/journal.pone.0275053)
Supplement: S1 File — (PDF) [file pone.0275053.s001.pdf]

## Supplementary material

*Supplementary Table 1 Study sample - organisation submissions included for analysis*

| | Organisation Type | Definition | Organisation Name | Count |
|---|---|---|---|---|
| **Industry Organisations** | Tobacco Industry | "Tobacco manufacturers, wholesale distributors and importers of tobacco products" (1) and extends to organisations that received funding, either directly or indirectly from the tobacco industry. | Imperial Brands Australasia<br>British American Tobacco (BAT)<br>Japan Tobacco International (JTI)<br>JUUL Labs (Altria – Philip Morris International)<br>Nicoventures Trading Limited (British American Tobacco)<br>Centre of Research Excellence: Indigenous Sovereignty & Smoking (PMI) (2,3) | 6 |
| | E-cigarette Industry | E-cigarette manufacturers, distributors, importers and "specialist" retailers | Antifun Limited TA Premium<br>Coastline Vapes<br>Cosmic<br>Easy As E-Cigs Ltd<br>Global Innovations Ltd<br>Hawke's Bay Vapour<br>Jubby's Juice Limited<br>Lion Labs<br>Mission Limited<br>NZVAPOR LIMITED<br>Te Wairua Limited (Vapour eyes)<br>VAPO<br>Vape Merchant<br>Vapourium<br>Vice Vape Co. | 15 |
| | | | **Total Industry Organisations** | **21** |
| **Health Organisations** | Non-government health organisations | Independent health-focused organisations | New Zealand Drug Foundation<br>Asthma and Respiratory Foundation (Te Ha Ora)<br>Cancer Society New Zealand<br>Heart Foundation of New Zealand<br>Lung Foundation New Zealand<br>Alcohol Healthwatch<br>ASH NZ - Action for Smokefree NZ<br>Stroke Foundation of New Zealand<br>End Smoking NZ | 9 |
| | Medical and Health Associations | Associations and representative groups of health and medical professionals | Public Health Association of New Zealand<br>New Zealand College Public Health Medicine<br>The Paediatric Society of New Zealand<br>The Royal Australasian College of Physicians<br>Royal Australasian College of Surgeons<br>Royal New Zealand College of General Practitioners<br>New Zealand Medical Association<br>New Zealand Nurses Organisation | 8 |
| | Research/Academic | Health research groups affiliated with recognised tertiary education providers. | ASPIRE 2025<br>Hapai Te Hauora Tapui Limited<br>National Institute for Health Innovation (NIHI)<br>Adolescent Health Research Group | 4 |
| | Health Service Providers | Organisations that deliver health services | Toi Te Ora Public Health (Lakes & Bay of Plenty Public Health Unit)<br>Nga Tai Ora - Public Health Northland<br>Auckland Regional Public Health Service<br>WellSouth Primary Health Network<br>Pegasus Health (Charitable) Ltd<br>Mahitahi Hauora PHE<br>Canterbury District Health Board<br>Hawke's Bay District Health Board<br>Waikato District Health Board<br>Whanganui District Health Board<br>Smokefree Canterbury<br>Smokefree Mid Canterbury<br>Smokefree Murihiku<br>Smokefree Otago<br>Takiri Mai te Ata Regional Stop Smoking Service | 15 |
| | | | **Total Health Organisations** | **36** |

*Supplementary Table 2 Frequently cited supporting evidence*

| Citation | Publication type | Count |
|---|---|---|
| **Frequently cited publications by health organisations** | | |
| Walker N, Parag V, Wong SF, Youdan B, Broughton B, Bullen C, et al. use of e-cigarettes and smoked tobacco in youth aged 14–15 years in New Zealand: findings from repeated cross-sectional studies (2014–19). Lancet Public Health. 2020 Jan;S2468266719302415 | Peer-reviewed Journal Article | 14 |
| National Academies of Science Engineering and Medicine. (2018) Public health consequences of E-Cigarettes. Washington, DC: The National Academies Press, 2018. | Peer-reviewed Book | 11 |
| Meernik, C., Baker, H. M., Kowitt, S. D., Ranney, L. M., & Goldstein, A. O. (2019). Impact of non-menthol flavours in e-cigarettes on perceptions and use: an updated systematic review. BMJ open, 9(10), e031598. | Peer-reviewed Journal Article | 10 |
| Gendall P, Hoek J. Role of flavours in vaping uptake and cessation among New Zealand smokers and non-smokers: a cross-sectional study. Tob Control | Peer-reviewed Journal Article | 9 |
| Bateman J, Robertson L, Marsh L, Thornley L, Hoek J. (2019) New Zealand tobacco retailers' understandings of and attitudes towards selling Electronic Nicotine Delivery Systems: a qualitative exploration. Tobacco Control. 2019:tobaccocontrol-2019-055173. | Peer-reviewed Journal Article | 9 |
| Zare, S., Nemati, M., & Zheng, Y. (2018). A systematic review of consumer preference for e-cigarette attributes: Flavor, nicotine strength, and type. PLoS One, 13(3). | Peer-reviewed Journal Article | 9 |
| Hoek J, Edwards R, Waa A, Wilson N, Thomson G. (2020) Public Health Expert Blog: Proposed Vaping Regulations for NZ: Strengths and Limitations. Posted Feb 24 2020. | Academic Blog | 8 |
| Ball, J., Fleming, T., Archer, D., & Sutcliffe, K. (2020) Youth19 – Vaping Fact Sheet. | Report | 8 |
| ASH New Zealand. (2019). Factsheet – E-cigarettes and vaping 2019. | Report | 8 |
| Marsh L, Doscher C, Robertson L. (2013). Characteristics of tobacco retailers in New Zealand. Health & Place 23 ( 2013) 165- 170. | Peer-reviewed Journal Article | 7 |
| **Frequently cited publications by industry** | | |
| Vaping in England: evidence update March 2020 [Internet]. [cited 2020 May 13]. | Report | 5 |
| Bates, C., Beaglehole, R., Laking, G., Sweanor, D., & Youdan, B. (2019). *A Surge Strategy for Smokefree Aotearoa 2025: The role and regulation of vaping and other low-risk smokefree nicotine products. Auckland:* ASH New Zealand and End Smoking New Zealand. | Report | 3 |
| Britton J, Arnott D, McNeill A, Hopkinson N, Tobacco Advisory Group of the Royal College of Physicians. Nicotine without smoke-putting electronic cigarettes in context. BMJ. 2016;353(8900488, bmj, 101090866):i1745. | Report | 3 |
| Smokefree Environments and Regulated Products (Vaping) Amendment Bill — Instruction to Health Committee - New Zealand Parliament [Internet]. [cited 2020 Mar 13]. | Bill | 3 |

| | | |
|---|---|---|
| Farsalinos KE, Romagna G, Tsiapras D, Kyrzopoulos S, Spyrou A, Voudris V. Impact of Flavour Variability on Electronic Cigarette Use Experience: An Internet Survey. Int J Environ Res Public Health. 2013 Dec;10(12):7272–82. | Peer-reviewed Journal Article | 2 |
| Ann McNeill, Leonie S Brose, Robert Calder, Linda Bauld, Debbie Robson. Evidence review of e-cigarettes and heated tobacco products 2018 [Internet]. Public Health England; 2018 p. 243. | Report | 2 |
| Russell C, McKeganey N, Dickson T, Nides N Mitchell Craig. Changing patterns of first e-cigarette flavor used and current flavors used by 20,836 adult frequent e-cigarette users in the USA. Harm Reduct J. 2018 Jun 28;15(1):33.(Tobacco Industry Research) | Peer-reviewed Journal Article | 2 |
| Walker N, Parag V, Wong SF, Youdan B, Broughton B, Bullen C, et al. use of e-cigarettes and smoked tobacco in youth aged 14–15 years in New Zealand: findings from repeated cross-sectional studies (2014–19). Lancet Public Health. 2020 Jan;S2468266719302415 | Peer-reviewed Journal Article | 2 |
| Use of e-cigarettes (vaporisers) among adults in Great Britain [Internet]. ASH UK; [cited 2020 May 21]. | Report | 2 |
| Science and Technology Committee. E-cigarettes Seventh Report of Session 2017–19 [Internet]. House of commons.; 2018 [cited 2020 May 22]. | Report | 2 |

*Supplementary Table 3 Statements and themes relating to frequently cited evidence – Youth e-cigarette use and smoking*

| STATEMENTS RELATING TO FREQUENTLY CITED EVIDENCE– YOUTH E-CIGARETTE USE AND SMOKING | | |
|---|---|---|
| | **THEME 1: SMOKING CESSATION FOCUS**<br>• e-cigarette use is supporting a reduction in youth smoking and/or<br>• e-cigarette prevalence among youth is negligible | |
| **PUBLICATION** | QUOTES | ORGANISATIONS |
| ***WALKER ET AL. 2020 (4)*** | *Indeed, in New Zealand, evidence suggests that e-cigarettes might be displacing smoking. A government funded cross-sectional study on the use of e-cigarettes and smoked tobacco in youth aged 14-15 years, found that" [t]he overall decline in smoking over the past 6 years in New Zealand youth suggests that e-cigarettes might be displacing smoking."* | Nicoventures Trading Limited (British American Tobacco) |
| | *"According to The Lancet, widespread teen vaping does not exist in New Zealand. In fact, vaping is displacing smoking among teens."* | Global Innovations Ltd |
| | *"The survey is completed by around 30,000 students each year, half the eligible year 10 population. The findings concluded that there was no vaping epidemic, and vaping may be displacing smoking in young people"*<br>*"For the vast majority of young people who have tried vaping, it is something they have tried once, or do less than monthly"* | ASH NZ - Action for Smokefree NZ |
| | *"We believe it is desirable for regulations to be set that are risk-proportionate, as evidence suggests that under current legislation, e-cigarettes may be displacing smoking in Aotearoa New Zealand's society"* | The Royal Australasian College of Physicians |
| ***ASH NZ, 2019 (5)*** | *A recent study found that only 0.8% of Year 10 vapers had never smoked previously, with the majority of minors who vape being former or already active smokers"* | COSMIC |
| | *"So far, no reputable epidemiologist or scientific evidence in New Zealand has found evidence of a youth vaping epidemic."* | ASH NZ - Action for Smokefree NZ |
| | *"In New Zealand, some young people vape. Fortunately, this seems to be largely confined to experimental use, with 37% of 14- and 15-year olds having ever tried vaping products, but only 3% using daily"*<br>*"Encouragingly, only 0.8% of 14- and 15-year olds who have never smoked are daily vapers"* | New Zealand Drug Foundation |
| | *"Data from the ASH year 10 survey (New Zealand's largest annual survey of year 10 students: https://www.ash.org.nz/) shows that the prevalence of daily smoking has halved in the last 10 years, from 5.5% in 2009, to 2.1% in 2019. Therefore, as a country we are achieving the smoking initiation goal recommended by the modelling"* | National Institute for Health Innovation (NIHI) |
| ***BALL ET AL. 2020 (6)*** | *"In March 2020, the youth 2000 survey published 2019 data on youth vaping and smoking. The factsheet stated that non-smokers vape, and many high school vapers are non-smokers. This claim is misleading and alarmist. In particular as it does not distinguish between experimentation, irregular use and dependence on nicotine"* | ASH NZ - Action for Smokefree NZ |
| | **THEME 2: YOUTH AND NON-SMOKER PROTECTION FOCUS**<br>• e-cigarette prevalence among youth is high, and/or<br>• youth smoking rates are increasing | |

| PUBLICATION | QUOTES | ORGANISATIONS |
|---|---|---|
| ***WALKER ET AL. 2020 (4)*** | *"A 2019 New Zealand school survey showed that 37.3% of 14-15-year olds reported they had tried vaping,"*<br>*"For the first time in 20 years, regular smoking prevalence in 14-15-year olds has increased rather than decreased in New Zealand (significant increases in non-Māori, non-Pacific students)."* | Alcohol Healthwatch |
| | *"Concerningly, the decline in cigarette smoking in adolescents has stalled or even reversed over the same period, particularly among Māori and students in low decile schools (Figs 1 & 2)".*<br>*"As shown in the Fig 1, regular smoking in Year 10 rangatahi Māori reached a low of 11% in 2015/16 and has since risen to 14% (2019)."*<br>*"Indeed, one possible explanation for the recent increase in smoking in Year 10 students from such communities (Fig 2) is that such transitioning from vaping to smoking is already occurring."* | Adolescent Health Research Group |
| | *"Data on adolescent vaping are available from the ASH Yr10 study. A recent paper reported a substantial increase in vaping from 2015 to 2019 with ever use increasing from 23% to 37%, regular (at least monthly) use from 3.5% to 12.0%, and daily use from 1.1% to 3.1%"* | ASPIRE2025 |
| | *"In the same study, more than one-half (58.5%) of Māori 14-15-year olds reported that they had tried vaping."* | Alcohol Healthwatch<br>Takiri Mai te Ata Regional Stop Smoking Service |
| | *"We are also concerned to see that between 2018 and 2019, regular smoking increased significantly in Māori Year 10 students, from 11.6 % to 13.6 %"* | Toi Te Ora Public Health (Public Health Unit) |
| | *"We are also concerned to see that between 2018 and 2019, regular smoking increased significantly in Māori Year 10 students, from 11.6 % to 13.6 %."* | Toi Te Ora Public Health (Public Health Unit)<br>Cancer Society New Zealand |
| | *"We are concerned about recent research that found vaping among young people has increased in New Zealand [(6)] whilst the trend in smoking decreasing in young people seems to have stalled. This calls into question the idea that vaping is displacing smoking. The alternative is that vaping is fuelling smoking. For this reason we must pursue the strongest regulations possible for both tobacco and vape products."* | Mahitahi Hauora PHE |
| | *"For the first time in 20 years, regular smoking prevalence in 14-15-year olds has increased rather than decreased in New Zealand."* | Alcohol Healthwatch<br>Public Health Association of New Zealand<br>Takiri Mai te Ata Regional Stop Smoking Service<br>WellSouth Primary Health Network |
| | *Despite vaping products being R18, in a 2019 New Zealand school survey 37.3% of 14- 15-year olds said they had tried vaping.* | WellSouth Primary Health Network<br>Smokefree Otago<br>Takiri Mai te Ata Regional Stop Smoking Service |
| | *"Despite vaping products being R18, in a 2019 New Zealand school survey 37.3% of 14- 15-year olds said they had tried vaping. This equates to more than 22,000 14 – 15 year olds who said they'd tried vaping. In the same study more than half (58.5%) of Māori 14-15-year olds had tried vaping."* | Public Health Association of New Zealand |
| | *"A recent article in Lancet Public Health2 has borne out these concerns based on ASH NZ Year 10 annual surveys – 31% of regular vapers and 21% of daily vapers had never smoked. In addition data from that paper and from published ASH NZ data show (Appendix) that as vaping has taken off among NZ young people, the rate of smoking decline has slowed, halted and then increased for the first time in two decades."* | The Paediatric Society of New Zealand |
| | *"The most recent data from 2019 shows an increase in regular smoking and vaping for Year 10 students, with a greater increase for Maori than non-Maori"* | Alcohol Healthwatch<br>WellSouth Primary Health Network |

| | | |
|---|---|---|
| | *"For the first time in 20 years, regular smoking prevalence in 14-15-year olds has increased rather than decreased in New Zealand."* | Alcohol Healthwatch<br>Public Health Association of New Zealand<br>Takiri Mai te Ata Regional Stop Smoking Service<br>WellSouth Primary Health Network |
| ***ASH NZ, 2019 (5)*** | *"There is growing evidence that people who have never smoked are taking up vaping"* | Auckland Regional Public Health Service |
| | *"The most recent data from 2019 shows an increase in regular smoking and vaping for Year 10 students, with a greater increase for Māori than non-Māori."* | Alcohol Healthwatch<br>WellSouth Primary Health Network |
| | *"We note that there is emerging evidence that the recent increase in vaping among school students (nearly 40 % had tried vaping in 2019, and 12 % were vaping regularly has been accompanied by an increase in regular youth smoking"* | Toi Te Ora Public Health (Public Health Unit)<br>Cancer Society New Zealand |
| | *"Due to aggressive industry marketing and easy access to these products over the last 18 months or so, there is now a large cohort of Year 10 (14-15-year olds) who have experimented with (37.3%) or regularly used (12%) e-cigarettes in 2019."* | Cancer Society New Zealand |
| | *"The most recent ASH Year 10 survey identified that there is high experimentation of vaping amongst youth and that there is an increase in the number of youth who are regular users of vape products"* | Nga Tai Ora - Public Health Northland |
| ***BALL ET AL. 2020 (6)*** | *"The Youth 19 survey includes 7700 students aged 13-18 years from 52 Auckland, Northland and Waikato schools surveyed in 2019, Ever (38%) and regular (at least monthly) use (10%) prevalences were very similar to the findings of the ASH Year 10 survey. At least weekly use prevalence was 6%. Regular and weekly use was greater among male and Māori and European students and students at more affluent (decile 8-10) schools. Interestingly, almost half (48%) of regular vapers had never smoked a cigarette."* | ASPIRE2025 |
| | *"The findings do not support the idea that young vapers are people who would otherwise be (or become) smokers (Ball et al, 2020). Rather they that show patterns of youth vaping are different from those of cigarette smoking. The findings suggest that vaping appeals to a wider range of young people."*<br>*"Importantly, we found many vapers had never smoked a cigarette. Two-thirds (65%) of those experimenting with vaping, nearly half (48%) of regular vapers, and about a third of weekly vapers reported they had never smoked."*<br>*"Interestingly, the strong social gradient seen with cigarette smoking in this age group was not apparent with vaping (Fig 3). Although students in low decile schools were more likely to have tried vaping, students in mid and high decile schools were more likely to be regular vapers"* | Adolescent Health Research Group |
| | *"A 2019 study found that 38% of Northland secondary school students have tried vaping, with 10% of students vaping regularly"* | Nga Tai Ora - Public Health Northland |
| | *"Significantly, sixty five percent of students who had ever vaped and forty eight percent of those who regularly vaped had never smoked cigarettes"* | Smokefree Canterbury |

*Supplementary Table 4 Statements and themes relating to frequently cited evidence - flavours*

| Statements relating to frequently cited evidence– flavours | | |
|---|---|---|
| | **Smoking cessation focus**<br>• flavours are important for smoking cessation and/or<br>• restrictions could have unintended consequences for smokers, e.g., relapse | |
| **Publication** | Quotes | Organisations |
| **Russell et al. (2018) (25)** | *"Those who previously smoked and switch to vaping are more likely to initiate with, transition to and/or use non-tobacco flavours and/or non-tobacco mint/menthol flavours."* | Auckland Regional Public Health Service |
| | *"Studies have shown that the majority of adult e-cigarette users use flavoured EVPs and there is evidence to suggest that flavoured EVPs improve transitioning from cigarette smoking to using EVPs, compared to non-flavoured EVPs"* | Imperial Brands Australasia |
| | *"The study is one of the largest surveys of e-cigarettes users ever conducted. The study found that: "[b]etween 2011 and 2016, the proportion of first END purchases that were flavored to taste like a fruit had almost doubled, while tobacco-flavored first END purchases had almost halved. These data suggest a transition in flavour preference at END use initiation over time, from tobacco to non-tobacco flavours, which is consistent with data from a US nationally representative survey that found both former-smoking exclusive END users and dual users reported significantly higher rates of current use of a non-tobacco-flavor –72.5% and 72.9%, respectively – compared to initiation."*<br>*"Based on the observed trend, the study concluded that restricting the availability of e-cigarette flavours could reduce adult smokers' interest in switching to e-cigarettes and raises the possibility that e-cigarette users could return to combustible tobacco products"* | Nicoventures Trading Limited (British American Tobacco) |
| **Farsalinos et al (2013) (7)** | *"Those who previously smoked and switch to vaping are more likely to initiate with, transition to and/or use non-tobacco flavours and/or non-tobacco mint/menthol flavours."* | Auckland Regional Public Health Service |
| | *"ASH UK's official advice is that the removal will likely cause some smokers to relapse"* | Imperial Brands Australasia |
| | *"The study concluded that "EC liquid flavourings play a major role in the overall experience of dedicated users and support the hypothesis that they are important contributors in reducing or eliminating smoking consumption."* | Nicoventures Trading Limited (British American Tobacco) |
| **Gendall et al. (2020) (8)** | *"Flavours appear to play an important role in cessation and are established in the New Zealand market with 47.8% of respondents to one survey using fruit flavour."* | Auckland Regional Public Health Service |
| | *"Research suggests over-regulation may encourage NGP users, particularly vapers, to concoct DIY flavours"* | Imperial Brands Australasia |
| | *"Flavour is an important part of the appeal of vaping as an alternate to smoking"* | New Zealand Drug Foundation |
| | **Youth and non-smoker focus**<br>• e-cigarette flavours increase appeal beyond smokers, (non-smokers/young people) and/or<br>• flavours decrease harm perceptions | |
| **Gendall et al. (2020) (8)** | *"New Zealand research has found flavours play a major role in vaping initiation for current smokers, former smokers and never smokers including young people.* | Alcohol Healthwatch |
| | *"New Zealand research found flavours play a major role in vaping initiation for people who currently smoke, formerly smoked and never smoked including young people. Fruit flavours were most popular among all three groups while people who have never smoked also liked confectionary/ sweets and lolly flavours"* | WellSouth Primary Health Network |

| | | |
|---|---|---|
| | *"New Zealand research found flavours play a major role in vaping initiation for current smokers, former smokers and never smokers including young people."* | Hawke's Bay District Health Board<br>Takiri Mai te Ata Regional Stop Smoking Service<br>Public Health Association of New Zealand |
| | *"Similarly, a survey of almost 700 NZ vapers and ex-vapers found that fruit flavours were the most popular, with tobacco, mint or menthol, and sweets/lolly flavours also popular"* | ASPIRE 2025 |
| | *"In 2019, a New Zealand online survey of 324 current vapers (vaped in the last 30 days) 83% of the 270 current vapers who also smoked tobacco, 77% of the 43 current vapers who were former smokers, and 82% of the 11 current vapers who had never- smoked, cited liking flavours as one of the main reasons why they vaped."* | National Institute for Health Innovation (NIHI) |
| | *"Fruit flavours were most popular among all three groups while people who have never smoked also liked confectionary/ sweets and lolly flavours"* | Takiri Mai te Ata Regional Stop Smoking Service<br>Public Health Association of New Zealand<br>Alcohol Healthwatch |
| **ZARE ET AL. (2018) (9)** | *Studies have concluded that flavours are important in making vaping products appealing to smokers, but that they also appeal to adolescents and young people"* | ASPIRE 2025 |
| | *"Adult smokers tend to prefer tobacco flavouring, while young people prefer non-tobacco flavoured e-cigarettes and overall prefer sweet, menthol and fruit flavours"* | Cancer Society New Zealand |
| | *"Flavours may be the most important reason for adolescents trying e - cigarettes."* | Takiri Mai te Ata Regional Stop Smoking Service<br>WellSouth Primary Health Network<br>Public Health Association of New Zealand<br>Smokefree Murihiku<br>Hawke's Bay District Health Board<br>Alcohol Healthwatch |
| | *"[our organisation] supports restricting flavours to R18 specialist stores as flavours may be the most important reason for adolescents trying e-cigarettes"* | Smokefree Otago |
| **MEERNIK ET AL. (2019) (10)** | *Studies have concluded that flavours are important in making vaping products appealing to smokers, but that they also appeal to adolescents and young people"* | ASPIRE 2025 |
| | *"Systematic reviews found flavours. increase the appeal, decrease the perception of harm and increase willingness to try ecigarettes"* | WellSouth Primary Health Network<br>Smokefree Murihiku<br>Takiri Mai te Ata Regional Stop Smoking Service<br>Public Health Association of New Zealand<br>Hawke's Bay District Health Board<br>Smokefree Otago<br>Alcohol Healthwatch<br>Cancer Society New Zealand |
| | *"It has been found in the United States, that sweet flavours for e-cigarettes increase product appeal, decrease product harm perceptions and increase willingness to use and initiation of e-cigarettes."* | The Royal Australasian College of Physicians |
| | *"Systematic reviews suggest that flavours increase product appeal, decrease harm perception, and may be the most important factor in young people trying e-cigarettes"* | Cancer Society New Zealand |
| **RUSSELL ET AL. (2018) (11)** | *"On the one hand, some argue that access to a wide-range of flavours enhances the appeal and effectiveness of vaping products, and thus the likelihood that smokers will use these products as complete substitutes for smoked tobacco (11) [...] However, while increasing the appeal of vaping products to smokers is likely to be beneficial, there are serious concerns about attributes that increase vaping products' appeal to young people."* | ASPIRE 2025 |
